# Positive Psychological Wellbeing Is Required for Online Self-Help Acceptance and Commitment Therapy for Chronic Pain to be Effective

**DOI:** 10.3389/fpsyg.2016.00353

**Published:** 2016-03-11

**Authors:** Hester R. Trompetter, Ernst T. Bohlmeijer, Sanne M. A. Lamers, Karlein M. G. Schreurs

**Affiliations:** ^1^Centre for eHealth and Wellbeing, Department of Psychology, Health and Technology, University of TwenteEnschede, Netherlands; ^2^Roessingh Research and DevelopmentEnschede, Netherlands

**Keywords:** chronic pain, moderator, predictor, psychological wellbeing, Acceptance and Commitment Therapy, web-based, online, resilience

## Abstract

The web-based delivery of psychosocial interventions is a promising treatment modality for people suffering from chronic pain, and other forms of physical and mental illness. Despite the promising findings of first studies, patients may vary in the benefits they draw from self-managing a full-blown web-based psychosocial treatment. We lack knowledge on moderators and predictors of change during web-based interventions that explain for whom web-based interventions are especially (in)effective. In this study, we primarily explored for which chronic pain patients web-based Acceptance and Commitment Therapy (ACT) was (in)effective during a large three-armed randomized controlled trial. Besides standard demographic, physical and psychosocial factors we focused on positive mental health. Data from 238 heterogeneously diagnosed chronic pain sufferers from the general Dutch population following either web-based ACT (*n* = 82), or one of two control conditions [web-based Expressive Writing (EW; *n* = 79) and Waiting List (WL; *n* = 77)] were analysed. ACT and EW both consisted of nine modules and lasted nine to 12 weeks. Exploratory linear regression analyses were performed using the PROCESS macro in SPSS. Pain interference at 3-month follow-up was predicted from baseline moderator (characteristics that influence the outcome of specific treatments in comparison to other treatments) and predictor (characteristics that influence outcome regardless of treatment) variables. The results showed that none of the demographic or physical characteristics moderated ACT treatment changes compared to both control conditions. The only significant moderator of change compared to both EW and WL was baseline psychological wellbeing, and pain intensity was a moderator of change compared to EW. Furthermore, higher pain interference, depression and anxiety, and also lower levels of emotional well-being predicted higher pain interference in daily life 6 months later. These results suggest that web-based self-help ACT may not be allocated to chronic pain sufferers experiencing low levels of mental resilience resources such as self-acceptance, goals in life, and environmental mastery. Other subgroups are identified that potentially need specific tailoring of (web-based) ACT. Emotional and psychological wellbeing should receive much more attention in subsequent studies on chronic pain and illness.

## Introduction

Chronic pain is a prevalent, disabling and difficult-to-treat condition that affects both individual pain sufferers and society (Breivik et al., 2006). Where biomedical oriented treatment modalities focus on pain removal, psychosocial treatments based on a cognitive behavioral framework try to effectively restore functioning and enhance pain management (Williams et al., 2012). The last decade has seen an expansion in studies exploring *web-based* delivery of psychosocial interventions for chronic pain and an additional, broad range of physical and mental health problem. First review studies indicate that web-based Cognitive Behavioural Therapies (CBT) are effective for chronic pain and other disorders (Cuijpers et al., 2010; Bender et al., 2011). Advantages that are associated with web-based psychosocial interventions are its cost- and time-effectiveness and its ability to reach physically disabled, stigmatized, or isolated patient groups. Furthermore, online interventions enable individuals to follow an intervention at their own pace (Andersson and Cuijpers, 2008). Even minimal improvements during self-help interventions that can be easily disseminated through the Internet to many individuals may contribute to alleviate the general disease burden of chronic pain and illness. Despite the promising findings of first studies, patients may vary in the benefits they draw from self-managing a full-blown web-based psychosocial treatment. At present, however, studies are lacking that specify for whom web-based cognitive behavioral interventions can be more or less profitable (Macea et al., 2010; Bender et al., 2011).

In general, to explore what, how, why and for whom psychosocial treatment does or does not work is a promising pathway to increase the effectiveness of psychosocial interventions for chronic pain and other physical and mental health problems (Kraemer et al., 2002; Morley and Keefe, 2007). Knowledge on moderators of change (‘for whom’) can inform future allocation of patients to treatment and guide tailoring of interventions to patient characteristics, thereby potentially enhancing both treatment effectiveness and efficiency (Morley et al., 2013). Such knowledge would be especially helpful in the area of chronic pain, as effects of both biomedical and psychosocial interventions are small to moderate and not all patients can be helped effectively at present (Turk et al., 2011; Eccleston et al., 2013). Unfortunately, there is a paucity of knowledge in this area. Factors that have been identified in *face-to-face* CBT for chronic pain to be negatively associated with treatment response include baseline levels of high psychological distress, low perceptions of pain control, high levels of negative thinking (e.g., catastrophizing) toward the pain, and stress (McCracken and Turk, 2002; Turner et al., 2007). No consistent relationships were found in previous CBT-studies between patient outcomes and demographic variables (McCracken and Turk, 2002).

The present study explores moderators (baseline characteristics that interact with treatment to affect outcome) and non-specific predictors (baseline characteristic that do not interact with treatment, but predict outcome regardless of treatment) of treatment change during a large, three-armed randomised controlled trial (RCT) on the efficacy of a guided, self-help web-based program based on Acceptance and Commitment Therapy (ACT) (Hayes et al., 2012; Trompetter et al., 2014). ACT is a distinct form of CBT that teaches pain patients to recognize and abandon unfruitful and narrowing attempts to avoid the pain experience itself and related thoughts and feelings (Hayes et al., 2006). Overall, therapeutic processes that are targeted in ACT – including pain acceptance, cognitive defusion and mindfulness – promote psychological flexibility, the ability to behave in accordance with personal, meaningful values from an open, accepting and present-moment stance toward the pain experience. ACT is an effective treatment for both chronic pain and a broader range of mental and physical health problems (Powers et al., 2009; Veehof et al., 2011; A-Tjak et al., 2015). Outcomes of the RCT generally showed small to moderate effects for the ACT-program Living with Pain compared to two (minimal intervention and waiting-list) control conditions in improving several disability-related processes and outcomes (Trompetter et al., 2014).

Of specific interest is *positive mental health* in addition to standard demographic, physical and psychosocial domain factors in chronic pain and psychosomatic research (Keyes, 2002). Positive mental health is a state of optimal mental functioning that consists of the aspects emotional, psychological and social wellbeing (Keyes, 2002). While emotional wellbeing relates to hedonic aspects of happiness, psychological wellbeing relates to eudemonic aspects of functioning that, for example, include feelings of personal growth and environmental mastery (Ryff, 1989, 2014). Social wellbeing pertains to feelings of social coherence, integration and social contribution (Keyes, 2002). Positive mental health and especially psychological wellbeing is related to resilience, the ability to maintain wellbeing despite life adversities such as enduring pain or to bounce back after adversities (Fava and Tomba, 2009; Ryff et al., 2012). We included a measure of positive mental health in our trial since the focus of ACT on commitment to personal goals that are intrinsically motivated, acceptance and mindfulness, is intrinsically and empirically supportive of increasing an rich, full and engaged life (Fledderus et al., 2012; Kashdan and Ciarrochi, 2013; Bohlmeijer et al., 2015). Also, psychological wellbeing is an underrepresented, but important and independent factor in relation to outcomes such as distress, chronic pain and physical frailty (Ruini et al., 2003; Schleicher et al., 2005; Gale et al., 2014).

Based on previous studies on face-to-face CBT for chronic pain, we predicted that psychosocial domain factors (depression, anxiety and positive mental health), and not physical domain factors (pain intensity, pain disability and pain interference) or demographic characteristics would function as moderators and predictors of change in pain interference in daily life during the RCT.

## Materials and Methods

### Participants and Procedure

The sample for the current study stems from the original sample in the RCT on the effectiveness of web-based ACT (Trompetter et al., 2014). The original RCT protocol was approved by the Dutch Medical-Ethical Review Board (METC, trial number NL38622.044.11), which operates under the Dutch Central Committee for Research involving human participants (CCMO). All subjects gave written informed consent in accordance with the Declaration of Helsinki. Participants were a heterogeneously diagnosed group of pain sufferers recruited from the general Dutch population through advertisements in Dutch newspapers and online patient platforms. Study inclusion criteria were (a) 18 years or older, (b) momentary pain intensity Numeric Rating Scale (11-point NRS) score > 4, (c) having pain for at least three days per week, (d) for at least 6 months. Exclusion criteria were partly based on the Hospital Anxiety and Depression Scale (HADS) (Zigmond and Snaith, 1983) and Psychological Inflexibility in Pain scale (PIPS) (Wicksell et al., 2010), and were (a) severe psychological distress (HADS > 24), (b) extremely low levels of psychological inflexibility (PIPS < 24), (c) current participation in another CBT-based treatment, (d) having no internet or e-mail address, (e) reading problems due to insufficient Dutch language skills or illiteracy, and (f) an unwillingness or inability to invest approximately 30 min per day. The primary reason for exclusion prior to randomization was severe psychological distress.

Participants in this study followed either the ACT-condition (*n* = 82) or were allocated to one of both control conditions, being either Expressive Writing (EW) (*n* = 79) or Waiting List (WL) (*n* = 77). EW was included as a control condition to control for general, non-specific effects (i.e., receiving attention from a counselor, working actively to reduce pain-related complaints). Small improvements in EW were expected as a large meta-analysis showed that EW has small effects on physical and mental health outcomes in chronic pain (Frattaroli, 2006). Those allocated to ACT or EW followed a 9-week web-based self-help program. WL-participants were not offered any intervention, but were free to access any other form of treatment. These participants could follow the ACT-intervention 6 months from baseline.

### Intervention

Each participant in ACT and EW received weekly minimal guidance and support on a fixed day of the week by trained clinical psychology students. In the ACT-condition, modules mainly consisted of text, metaphors and exercises based on the six ACT-therapeutic processes (pain acceptance/experiential avoidance, cognitive defusion, self-as-context, present-moment awareness, values and committed action) (Hayes et al., 2012). Two extra modules were included that did not explore ACT-processes, but focused on psycho-education regarding chronic pain (first module) and communicating about pain complaints with one’s social context (eight’ module). Following the first module on psychoeducation, the next four modules primarily explored favorite ways to experiential avoid pain, and explored acceptance of pain as an alternative strategy. Simultaneously, participants explored their values and subsequent goals in different life domains. The following two modules mainly explained and explored the two ACT-processes cognitive defusion and self-as-context, to learn to relate differently to oneself, one’s thinking states and one’s context. The final module again focused on committed action, and it was explored how one would cope with setbacks and failure in the long term. Participants were encouraged to download new mindfulness exercises weekly (e.g., ‘body scan,’ ‘breathing toward pain’ or ‘observe your thinking’), and practice mindfulness daily for 10–15 min. Participants were advised to spend approximately 30 min each day, or 3 h per week in total, on the course. In EW, the general assignment was to emotionally disclose (write) on a regular basis about experiences and emotions either related to chronic pain or to other situations. These emotions could be either negative or positive, depending on specific weekly assignments. Additionally, each module started with some short psycho-education about emotions and emotion regulation. Participants were asked to invest 2 h or more per week, or 15 min per day on the course. In both ACT and EW, participants could keep an online diary. The average time-investment was self-assessed at multiple times throughout the course. 48% and 47% of participants in ACT and EW respectively adhered to the intervention, which meant they both completed the intervention and invested the advised amount of time interacting with the course [*adherence* is the extent to which individuals experience the content of an intervention. This is different from *drop-out*, which refers to the number of people who did not follow the research protocol (i.e., did not fill in questionnaires; Kelders et al., 2012)].

### Measures

The primary outcome was measured at 3-month follow-up, 6 months after baseline assessment (T1). All other measures functioned as possible moderators/predictors of change and were assessed at baseline, prior to randomization (T0).

#### Outcome

##### Pain interference in daily life

The Multidimensional Pain Inventory (MPI), subscale *pain interference* consists of nine items and measures the degree to which pain interferes with different life domains, such as work, household work and social activities (Kerns et al., 1985). Higher scores indicate more pain interference (range 0–54). Internal consistency in the present study was at baseline α = 0.87, at T1 α = 0.89.

#### Moderator/Predictors

##### Demographic variables

Demographic variables that were assessed as possible moderators/predictors were age, gender, educational level, employment status, and duration of pain complaints.

##### Pain intensity

Pain intensity was measured with a 11-point Numeric Rating Scale (NRS), ranging from ‘no pain’ (0) to ‘pain as bad as you can imagine’ (10). Item formulation and response categories were consistent with IMMPACT recommendations on core outcome measures in chronic pain research (Dworkin et al., 2005).

##### Pain disability

The Pain Disability Index (PDI) (Pollard, 1984) consists of seven items and assesses the degree to which chronic pain disables a person from performing daily activities, such as work, household responsibilities and recreational activities. Total scores range from 7 to 70, with higher scores indicating more pain disability. Internal consistency in the current study at baseline was α = 0.82.

##### Psychological distress

The HADS (Zigmond and Snaith, 1983) consists of 14 items. The scale measures the presence and severity of symptoms regarding anxiety (seven items) and depression (seven items). In this study the both subscales were used, with sum scores for each scale ranging from 0–21. Higher scores indicate more anxiety or depression. Internal consistency in the present study at baseline was at α = 0.73 (anxiety) and α = 0.79 (depression).

##### Positive mental health

The Mental Health Continuum-Short Form (MHC-SF) (Keyes, 2002) consists of 14 items that measure three dimensions of positive mental health. Participants rate their frequency of feelings over the past month. Dimensions are *emotional wellbeing*, pertaining to positive feelings, happiness and satisfaction with life (three items) (score range 3–18); *psychological wellbeing*, pertaining to aspects of positive psychological functioning, such as autonomy, environmental mastery and personal growth (six items) (score range 6–36); and *social wellbeing*, pertaining to feelings of positive functioning in community life (five items) (score range 5–30). The MHC items did not show differential item functioning in a sample of individuals suffering from physical diseases compared to a healthy subsample (Lamers et al., 2012b). The total scale and all subscales are analyzed separately in this study. In general, higher scores indicate more wellbeing. Internal consistency in the current study at baseline was α = 0.91 (total MHC), α = 0.85 (emotional wellbeing), α = 0.82 (psychological wellbeing) and α = 0.73 (social wellbeing).

### Statistical Analyses

There were no missing data at T0. Missing data at T1 (29.8%) were imputed using the Expectation Maximization (EM) Algorithm (Dempster et al., 1977). Prior to main analyses, independent sample *t*-tests and χ^2^-tests were applied to determine if there were significant differences in all potential moderator/predictor variables at T0 between ACT and both control conditions.

In performing exploratory analyses, we followed steps taken by Turner et al. (2007) in a well-regarded study on moderators and predictors of change during CBT for chronic pain (Morley and Keefe, 2007). Pain interference in daily life at 3-month follow-up, as measured with the MPI interference subscale, was used as indicator of treatment effect. To determine if selected moderator/predictor variables functioned as moderators or predictors of change in MPI interference, linear regression models were applied using the PROCESS macro for SPSS (Hayes, 2013). All tests were two-tailed. Thirteen moderator/predictor variables were assessed, including age, gender, educational level, employment status, pain duration, pain intensity (NRS), pain disability (PDI), pain interference (MPI subscale), depression (HADS), anxiety (HADS), and emotional, psychological and social well-being (MHC). For demographic moderators/predictors, dummy variables were created for gender (male = 1, female = 0), employment status (working full/parttime = 1, other = 0), and duration of pain complaints (>5 years = 1, <5 years = 0). Educational level was divided into three groups (low, medium and high). During the analyses, each potential moderator/predictor was grand mean centered to reduce possible scaling problems and multicollinearity (Aiken and West, 1991). In the regression models, the MPI-interference score at T1 was entered as the dependent variable. The dummy variable representing Treatment (web-based ACT = 1, WL = 0 or EW = 0), the centered potential moderator/predictor, and the Treatment by centered moderator/predictor interaction variable were entered as independent variables. To control for baseline variation in outcome scores, the MPI interference score at T0 was added as independent variable to the model in the same step as all other independent variables. Analyses were performed separately for ACT compared to EW, and ACT compared to WL.

In the presence of a significant interaction effect the variable in concern was interpreted as being a moderator of change. In case the interaction effect was not significant but the main effect for the variable was, a variable was interpreted as being a predictor of change. Moderators are baseline characteristics that interact with treatment to affect outcome, meaning that patient improvement depends on the value on the moderator variable. When a variable is not a moderator, it is possibly a non-specific predictor of change. Non-specific predictors do not interact with treatment but predict later scores on outcomes for all participants. Both moderators and predictors of change should be measured prior to treatment randomization (Turner et al., 2007; Pincus et al., 2011). Overall, significance of the moderators and predictors was interpreted at *p* < 0.05. Although the number of tests performed could call for a restriction on the borderline *p*-value, the *p*-value was not adjusted as such given the exploratory nature of this study. In case of significant interactions, simple slopes for mean, -1 and +1 standard deviation moderator values as calculated in PROCESS were interpreted, as were outcomes of the Johnson-Neyman technique (Johnson and Fay, 1950; Hayes, 2013). This latter method derives a zone of significance, thereby identifying exact cut-off values of the moderator for which web-based ACT was (not) more effective compared to control conditions.

## Results

Outcomes of independent sample *t*-tests and χ^2^-tests revealed there were no significant differences at T0 between ACT and both control conditions on all included potential moderator/predictor variables, although the difference between ACT and WL in the percentage of people working full/part-time reached marginal significance, with ACT participants working full/part-time more often than WL participants [χ^2^(1) = 3.439, *p* = 0.064].

A large proportion of participants were highly educated (44.1%), female (76.0%) pain sufferers with an average age of 52.80 years (*SD* = 12.37). More than half of the participants suffered from pain complaints for more than 5 years (63.0%), and almost all participants (93%) reported pain on a daily basis. Most prevalent diagnoses were fibromyalgia (20.2%), back complaints (12.7%), rheumatic diseases (9.7%), neuropathic complaints (8.8%), and other joint complaints (8.4%). An overview of demographic characteristics and baseline scores on all measures can be found in **Table 1**.

**Table 1:** Baseline characteristics of participants in ACT and both control conditions.

	ACT (*n* = 82)	EW (*n* = 79)	WL (*n* = 77)
**Demographic characteristics**			
Mean age, years (SD)	52.9 (13.3)	52.3 (11.8)	53.2 (12.0)
Female gender (%)	76.8	75.9	75.3
Education (%)			
Low	19.5	19.0	22.1
Intermediate	35.4	36.7	35.0
High	45.1	44.3	42.9
Working full-/part-time (%)	42.7	48.1	28.6
Pain duration >5 years (%)	58.5	69.6	61.0
Diagnosis			
None	14.6	17.7	19.5
Back complaints	9.8	13.9	14.3
Fibromyalgia	15.9	29.1	15.6
Joint complaints	8.5	7.6	9.1
Rheumatic disease	9.8	7.6	11.7
Neuropathic complaints	11.0	6.3	9.1
Other	30.5	20.8	20.7
**Physical domain measures**			
Mean MPI Interference (SD)	32.3 (9.8)	32.2 (9.8)	33.3 (9.8)
Mean Pain intensity (SD)	6.3 (1.8)	6.1 (1.6)	6.2 (1.6)
Mean Pain Disability (SD)	36.0 (12.7)	36.4 (12.0)	36.1 (12.7)
**Psychosocial domain measures**			
Mean HADS depression (SD)	6.1 (3.5)	6.5 (3.5)	6.1 (3.2)
Mean HADS anxiety (SD)	7.2 (3.1)	7.5 (3.2)	6.9 (3.4)
Mean MHC emotional (SD)	12.4 (3.1)	12.1 (2.9)	11.1 (3.2)
Mean MHC psychological (SD)	23.9 (5.7)	23.9 (5.8)	22.8 (6.4)
Mean MHC social (SD)	16.2 (4.9)	16.2 (5.1)	16.0 (4.6)

*ACT, Acceptance and Commitment Therapy; EW, Expressive Writing; WL, Waiting List; MPI, Multidimensional Pain Inventory; HADS, Hospital Anxiety and Depression Scale; PDI, Pain Disability Index; MHC, Mental Health Continuum.*

### Moderators of Changes in MPI Interference

**FIGURE 1:**
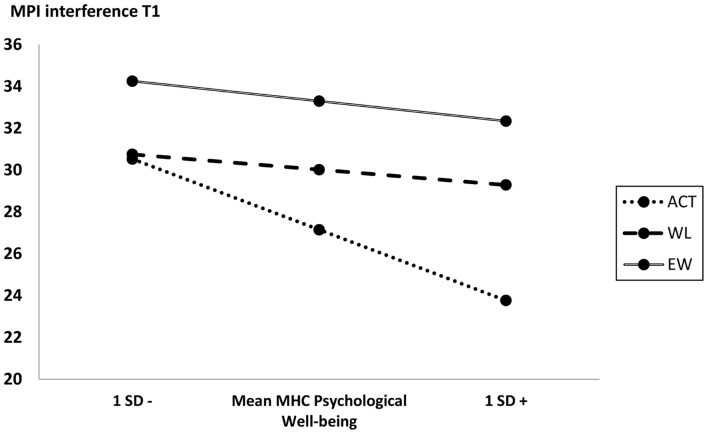
**Pain interference outcome scores at different baseline values of moderator MHC Psychological Wellbeing for ACT compared to both control conditions**.

Outcomes of interaction tests for all 13 potential moderators can be found in **Table 2**. No significant interaction effects on MPI interference at 3-month follow-up were present for any of the demographic variables. Of the remaining measures, the only interaction effect that reached significance compared to both control conditions was MHC Psychological wellbeing (vs. EW: *b* = -0.424, *p* = 0.035; vs. WL: *b* = -0.419, *p* = 0.022). A visual representation of the outcomes of simple slope analyses for mean scores, and scores one standard deviation below and above the mean value, are displayed in **Figure 1**. Web-based ACT was no more effective than WL in changing MPI interference for those scoring one standard deviation below mean (effect MPI interference T1 ACT vs. WL = 0.323, *p* = 0.837). More specifically, an interpretation of the output of the Johnson-Neyman technique showed that the MHC Psychological wellbeing cut-off score for reaching significant effects of ACT compared to WL was 23.57. ACT was more effective in changing the primary outcome MPI interference than WL for those in the highest 51% of MHC scores. Compared to control condition EW, the MHC Psychological Wellbeing cut-off score for reaching significant effects of ACT was 16.97. ACT was more effective in changing MPI interference than EW for those in the highest 88.2% of MHC scores.

**Table 2:** Interaction effect outcomes of linear regression models to assess possible moderators of change in MPI interference.

	ACT vs. EW			ACT vs. WL		
	*b*	95% CI	*p*-value	*b*	95% CI	*p*-value
**Demographic characteristics**						
Age	-0.152	-0.37; 0.07	0.172	0.804	-4.56; 6.18	0.768
Gender	-2.422	-9.10; 4.25	0.475	-0.003	-0.18; 0.18	0.978
Educational level	2.19	-1.39; 5.77	0.228	-0.110	-3.07; 2.85	0.941
Employment status	3.018	-2.00; 8.04	0.237	-2.788	-7.59; 2.02	0.253
Pain duration	0.878	-3.85; 6.61	0.763	2.289	-2.35; 6.93	0.332
**Physical domain measures**						
Pain intensity	-2.018	-3.36; -0.68	0.003	-0.371	-1.74; 1.00	0.594
Pain disability (PDI)	-0.179	-0.37; 0.02	0.073	-0.161	-0.34; 0.01	0.071
Pain interference (MPI)	-0.077	-0.37; 0.22	0.606	-0.135	-0.37; 0.10	0.251
**Psychosocial domain measures**						
Depression (HADS)	0.263	-0.40; 0.92	0.431	0.169	-0.46; 0.80	0.599
Anxiety (HADS)	0.254	-0.51; 1.01	0.510	0.732	-0.04; 1.50	0.063
Emotional wellbeing (MHC)	-0.712	-1.50; 0.07	0.074	-0.525	-1.22; 0.17	0.138
Psychological wellbeing (MHC)	-0.424	-0.82; -0.03	0.035	-0.419	-0.78; -0.06	0.022
Social wellbeing (MHC)	-0.460	-0.99; 0.07	0.128	-0.451	-0.95; 0.05	0.078

*95% CI, 95% confidence interval.*

None of the measures representing the physical domain, being pain intensity, PDI and MPI interference, showed significant interaction effects compared to WL. However, a significant moderation effect existed for ACT compared to EW alone on pain intensity (NRS) (*b* = -2.018, *p* = 0.003). An inspection of the output of the Johnson-Neyman technique indicated that ACT was more effective than EW for those individuals having the highest 85.1% scores on pain intensity (NRS) at baseline. The corresponding cut-off score was 4.61.

### Predictors of Change in MPI Interference

Outcomes regarding non-specific predictor analyses can be found in **Table 3**. As was the case for moderator analyses, none of the demographic characteristics were significantly associated with MPI interference at 3-month follow-up, and neither were baseline PDI and pain intensity. T0 measures that were significantly associated with MPI interference 6 months later were similar for both sets of analyses (ACT compared to EW and ACT compared to WL). Significant predictors were MPI interference (vs. EW: *b* = 0.732, *p* < 0.001, vs. WL: *b* = 0.760, *p* < 0.001), HADS depression (vs. EW: *b* = 0.632, *p* < 0.001, vs. WL: *b* = 0.628, *p* < 0.001), HADS anxiety (vs. EW: *b* = 0.806, *p* < 0.001, vs. WL: *b* = 0.529, *p* = 0.013) and MHC Emotional wellbeing (vs. EW: *b* = -0.554, *p* = 0.007, vs. WL: *b* = -0.627, *p* = 0.001).

**Table 3:** Main effect outcomes of linear regression models to assess possible predictors of change in MPI interference.

	ACT vs. EW			ACT vs. WL		
	*b*	95% CI	*p*-value	*b*	95% CI	*p*-value
**Demographic characteristics**						
Age	0.012	-0.10; 0.12	0.830	-0.061	-0.15; 0.03	0.183
Gender	2.997	-0.34; 6.33	0.078	1.412	-1.27; 4.11	0.299
Educational level	-1.054	-2.85; 0.74	0.247	0.084	-1.40; 1.56	0.911
Employment status	-0.928	-3.53; 1.67	0.482	2.006	-0.383; 4.39	0.099
Pain duration	0.369	-2.45; 3.19	0.796	-0.332	-2.65; 1.99	0.778
**Physical domain measures**						
Pain intensity	0.344	-0.44; 1.13	0.388	-0.570	-1.30; 0.15	0.118
Pain disability (PDI)	0.009	-0.20; 0.22	0.931	-0.054	-0.18; 0.08	0.420
Pain interference (MPI)	0.732	0.59; 0.88	<0.001	0.760	0.64; 0.88	<0.001
**Psychosocial domain measures**						
Depression (HADS)	0.632	0.22; 1.04	0.003	0.628	0.27; 0.99	0.001
Anxiety (HADS)	0.806	0.37; 1.25	<0.001	0.529	0.11; 0.95	0.013
Emotional wellbeing (MHC)	-0.554	-0.96; 0.15	0.007	-0.627	-0.99; -0.26	0.001
Psychological wellbeing (MHC)	-0.384	-0.59; -0.18	<0.001	-0.377	-0.57; -0.19	<0.001
Social wellbeing (MHC)	-0.205	-0.47; 0.06	0.128	-0.197	-0.44; 0.05	0.117

*95% CI, 95% confidence interval.*

## Discussion

The present study explored moderators and predictors of treatment change during a previously evaluated RCT on the efficacy of a guided, self-help web-based program based on ACT in chronic pain patients (Trompetter et al., 2014). Compared to both control conditions neither demographic nor physical domain factors prospectively predicted or moderated pain interference in daily life after 6 months. Despite variable findings in individual studies, this is in line with knowledge on predictors of face-to-face CBT treatment effects (McCracken and Turk, 2002). Importantly, the only existing moderator compared to both control conditions was psychological wellbeing as a central aspect of positive mental health and optimal human functioning (Keyes, 2002).

Emotional and psychological wellbeing are highly relevant factors that function independent from vulnerabilities and distress in predicting mental and physical illness (Ruini et al., 2003; Steptoe et al., 2009; Boehm and Kubzansky, 2012; Lamers et al., 2012a). This study suggests that psychological wellbeing is also relevant for allocation of treatment. Self-managing a challenging intervention that requires the transformation of cognitive-behavioral patterns that narrowed effective living for a prolonged period of time, could simply be too much for individuals lacking psychological resources. This process could evolve, for example, through a lack of feelings of environmental mastery, personal growth and positive social relations. Among other things, these processes relate to the feeling that oneself is able to develop new attitudes and behaviors, a sense of control over the external world, and the feeling that one is supported by significant others (Ryff, 1989, 2014; Fava and Tomba, 2009). Practically, these results indicate that web-based ACT should perhaps not be allocated to those experiencing low positive psychological functioning at baseline. A primary task for future web-based trials is to examine if aspects of resilience and psychological wellbeing recurrently function as moderators of treatment change for pain and other physical and mental health problems.

The design of psychosocial interventions that aim at enhancing resilience and psychological wellbeing provides interesting and perhaps necessary treatment opportunities for chronic pain and illness. Wellbeing Therapy (WBT) is a primary example of an effective, positive intervention designed explicitly to complement CBT that improves psychological wellbeing and prevents relapse for depression and anxiety disorders (Fava et al., 1998, 2004, 2005). Such an increase of psychological wellbeing to be able to bounce back from highly frequent and intense moments of distress can be highly relevant for those suffering from chronic pain and illness. We suggest future study explores the efficacy of the parallel application of resilience-based treatments such as WBT in addition to standard psychosocial treatments aimed at reducing pain-related complaints. The increase of effective adaptation and normal functioning in the face of chronic pain might help to overcome the modest effects of current chronic pain treatment (Turk et al., 2011; Eccleston et al., 2013).

Two other findings deserve exploration. First, a further interpretation of moderator findings indicates that EW might not work so well when high in pain intensity. This explains outcomes from a range of studies indicating that EW has mixed and at best, modest, benefits for people suffering from chronic pain (e.g., Lumley et al., 2013), while it seems more effective for mild and major depression (e.g., Gortner et al., 2006). Emotional disclosure can be an unsettling experience that can instigate more pain and negative mood in those suffering from chronic pain. Although not the primary target of our study, these findings can possible fuel further study on EW in chronic pain. Additionally, several non-specific predictor of change were identified. Higher baseline levels of depression, anxiety and pain interference in daily life, and lower levels of emotional wellbeing, were prospectively and generically related to higher levels of pain interference. Practically, this knowledge can be used to further explore if specific tailoring of web-based ACT and other web-based interventions toward these characteristics is helpful. Applying more intensive therapist guidance and monitoring for specific individuals are examples of tailoring opportunities. Also, the application of persuasive technology in developing web-based interventions offers interesting future venues for the future (Kelders et al., 2012).

An important limitation to this study is that the RCT protocol of this study was not powered *a priori* for the application of moderator analyses. Therefore, analyses were *post-hoc* and exploratory, and should be interpreted accordingly. It might be that the number of participants available to perform moderator analyses was not sufficient to indicate other potential relevant moderators of change in addition the moderators we identified. Nevertheless, our study pertains to methodological requirements of exploratory moderators studies (Turner et al., 2007; Pincus et al., 2011), and highlighted several interesting outcomes. Another limitation is that we produced specific cut-off scores to exemplify for whom self-help ACT seems specifically (in)effective. This is the first efficacy trial to produce cut-off scores, which are therefore not readily transferable to clinical practice. However, we believe that the production of our cut-off scores is one step forward to translating scientific output into useful applications for practice.

Overall, this study was the first to assess moderators and predictors of change during web-based psychosocial treatment for chronic pain. This resulted in relevant insights on the future allocation to pain sufferers of the ‘Living with Pain’ program in specific, and other web-based psychosocial interventions for pain and the broader range of physical and mental health disorders in general. Illuminating theoretical insights were gathered regarding ACT theory (Hayes et al., 2012) and findings revealed that, broadly, moderators of change for web-based ACT treatment seem to follow similar patterns as in face-to-face CBT. We hope that future studies use these outcomes as a springboard for further study. Of all topics discussed, a focus on psychological wellbeing and resilience seem most promising to further increase effective and efficient intervention for chronic pain and illness in the future.

## Author Contributions

HT designed the study, performed acquisition, analysis and interpretation of the data, and drafted the manuscript. EB was involved in the conception of the study, data interpretation and writing. SL contributed to data interpretation and writing of the manuscript. KS was responsible for conception of the study, supervised acquisition and analysis of data, and contributed to data interpretation and writing of the manuscript. All authors contributed to critical revisions of the manuscript and approved the final version of the manuscript.

## Conflict of Interest Statement

The authors declare that the research was conducted in the absence of any commercial or financial relationships that could be construed as a potential conflict of interest.
